# The Dental Team: An Additional Resource for Delivering Vaccinations

**DOI:** 10.3389/fmed.2020.606242

**Published:** 2020-11-06

**Authors:** Stefan Serban, Zhain Mustufvi, Jing Kang, Sally Eapen Simon, Siobhan Grant, Gail Douglas

**Affiliations:** ^1^School of Dentistry, University of Leeds, Leeds, United Kingdom; ^2^Public Health England, North East and Yorkshire Regional Office, Leeds, United Kingdom

**Keywords:** COVID-19, coronavirus, dentistry, dental team, vaccinations, influenza, flu, immunisation

## Introduction

Influenza is regarded by the World Health Organization (WHO) as a “serious global health threat” and is responsible for a significant number of deaths worldwide (1, 2). Influenza vaccines are usually delivered in primary care physician practices as well as in pharmacy settings. Dental practices are generally not seen as routine providers of influenza vaccines, although certain countries have been recently starting to develop frameworks to facilitate this (3, 4). Potential workforce capacity gaps, expansion of the eligible cohorts, requirement to drive improvements in uptake, and to reduce inequalities support the rationale for members of the dental team to join the vaccinator workforce. We propose a few potential models, some of which are currently being piloted in certain areas of the United Kingdom, and suggest that the members of the dental team might be ideally placed to contribute to national immunization programs.

The coronavirus pandemic has had a devastating effect on people's lives, pushing the limits of healthcare systems and placing enormous economic and political pressures on governments. As with other pandemics throughout history it has also highlighted the vulnerabilities of our society and forced us to reflect on our values and priorities, compelling us to develop new ways of delivering care, adapted to the new realities of the world around us (5). Face to face consultations became the exceptions and remote consultations the new norm (6). Thousands of clinical and non-clinical staff have been redeployed from their usual workplace into new settings to support the pandemic response (7).

In several countries around the world, the multi-skilled dental workforce has demonstrated that dentistry is ready to support the national health systems response in a time of emergency (8, 9). Members of the dental team were redeployed based on their set of competencies into various roles including medical history taking, phlebotomy, cannulation, suturing, and others (10). A properly trained and indemnified dental workforce would be ideally placed to support the delivery of the flu vaccination program for the next season (11). In 2009, during the H1N1 (swine flu) pandemic a precedent has been established when certain US states commissioned dentists to administer flu vaccines in order to increase capacity for delivery and meet the increased demand in a short time frame (12, 13).

## Delivery Models

The delivery models for seasonal flu vaccinations and for school vaccination programs vary between different healthcare systems around the globe. The next section presents some of the models that are currently being explored in the UK. These could be adapted for different healthcare settings based on the existing structures and processes developed nationally (14). The members of the dental team could support the delivery of the seasonal influenza vaccine, as well as other immunization programs such as the childhood vaccination program and potentially the large-scale delivery of COVID-19 vaccines, should a safe and effective vaccine become available in the future.

### Flu Vaccinations

Every year, the arrival of autumn means also the beginning of the flu season. Seasonal influenza has been a constant challenge for healthcare systems across the world and it is estimated to be responsible for nearly 400,000 deaths every year world-wide (2).

The uptake of influenza vaccines varies between different countries. The most accurate and comparable data is on vaccination rates for people over the age of 65. These rates present large variations from 10.2% in Estonia to 85.1% in South Korea (15). This year, national health systems are facing the risk of a dual threat created by seasonal influenza and the potential resurgence of COVID-19, which could have devastating effects especially in the high-risk groups. Certain governments have recognized this threat and established a set of preventative measures. For example, the UK Government has decided to expand the eligibility criteria for influenza vaccinations to include additional groups in the national immunization schemes. These additional groups consist of all people over the age of 50, people who are shielding from COVID-19 and their households, as well as secondary school children in England. This represents an estimated total of 30 million people, which represent an increase of 5 million people compared to the previous year (16, 17). This broadening of the eligibility criteria would lead to operational and logistical challenges for delivery in a timely manner. Expanding the workforce capacity to include the members of the dental team could attenuate some of these challenges.

The following models might serve as potential avenues that could be explored by policy makers in different countries.

#### Provision of Additional Personnel for Local Delivery Programs

Additional health protection measures have been imposed by COVID-19, such as dental surgery fallow time after an aerosol generating procedure and increased decontamination demands (18, 19). As a result, some dental teams may be utilizing less personnel and therefore these staff members could join existing immunization teams to deliver vaccinations in various settings such as schools, primary care, and specially allocated community settings. This would increase the available workforce capacity to deliver vaccinations in a short time frame. This is an important advantage as to maximize protection for the entire flu season, it would be essential to cover as many eligible people as possible as soon as the vaccines become available in the first few months of the season (20, 21). The most cost-effective solution would be to utilize dental nurses or hygienists/therapist to support this model however we believe that the option to administer vaccinations should be available to all members of the dental team as long as they are properly trained, competent, and indemnified.

#### Use of Premises

Dental surgeries represent a large network of clinical settings that are known and easily accessible to the public. Evidence suggests that certain cohorts of patients visit the dental surgery more frequently than their general physician (22). Larger dental surgeries are likely to have capacity in terms of surgery or floor space that could be used temporarily by immunization teams. Dental practices are also well-equipped to deal with rare but serious adverse reactions.

#### One-Stop Shop

Patients could choose to receive their influenza vaccine at the same time as their dental appointment. This model would support the principle of reduction of the number of contacts between patients and healthcare settings and reduce the risks associated with multiple journeys, especially in more rural areas where traveling longer distances is often required. It could be done both opportunistically as well as in a targeted manner for specific cohorts. This approach has already been successfully implemented in certain states in the US (13).

### Childhood Immunization Programs

Besides helping the delivery of influenza vaccines, members of the dental team could be deployed to support the catch-up initiatives for childhood immunization programs. In several countries around the world, the COVID-19 pandemic has led to school closures for a significant amount of time and this has created a backlog in terms of delivering immunization programs for school aged children with the increased risk of community outbreaks (23).

### COVID-19 Vaccinations

It is believed that a COVID-19 vaccine would be the best way to speed up the economic recovery and protect public health from the damage caused by the pandemic. The race to develop a safe and effective coronavirus vaccine has demonstrated the need for innovative thinking and collaboration between researchers from different scientific disciplines (24). The success or failure of any large-scale immunization program depends on the uptake of the vaccines by the majority of the population and it will raise significant logistic challenges (25, 26). If the above-mentioned models for flu vaccination could be piloted and evaluated, the lessons learned from these initiatives could inform the planning and delivery of a large-scale COVID-19 mass immunization program, with a vaccinator workforce which included dental team members.

## Discussion

Any of the above models would need to take into consideration certain factors such as:

- Approval from the regulatory bodies of dental care providers and professional indemnity providers;
- Approval from national regulatory agencies for medicines;
- Information governance and access to clinical data including patients' full medical history and robust intra-operability between data systems across dental and medical systems;
- Clear funding mechanism.

Research into the effectiveness, safety and cost-effectiveness of these interventions should be encouraged in order to inform future evidence-based decisions. Any service evaluation should also include a qualitative component for capturing the experiences of the new dental vaccinators as well the views of the existing vaccinating teams and patients.

It has become clear that the coronavirus pandemic highlighted the stark inequalities in our society. It was marked by a strong socioeconomic gradient, affecting disproportionately the most vulnerable groups. Public health measures such as prevention and health promotion are the among the key interventions to reduce the impact of inequalities (27, 28). Dental leaders have long been advocating the idea of “putting the mouth back in the body” (29, 30). There is a short window of opportunity, before the beginning of the flu season, to explore the untapped resource of the dental workforce in supporting the delivery of an enhanced immunization program. There is no better time to consider innovative solutions aimed to challenge the traditional boundaries between the different parts of the healthcare system (31). This is a crucial time to reconsider the segregated models of the past and move toward more integration of resources to support the uptake of one of the largest flu vaccination programs to date (32). Seasonal influenza has the potential to place additional strains on the health and social care systems which were already hard-hit by the coronavirus pandemic. This is the time to design preventative measures to mitigate the dual threat caused by influenza and COVID-19 and to think holistically about the resources available in the healthcare system. Engaging the dental team for delivering influenza vaccinations could further strengthen the collaboration between medicine and dentistry and is likely to set an important precedent to meet the demands of future public health crisis.

## Author Contributions

SS prepared the first draft of the manuscript. All the other co-authors contributed with comments and approved the final version.

## Conflict of Interest

The authors declare that the research was conducted in the absence of any commercial or financial relationships that could be construed as a potential conflict of interest.
