# Corrigendum: The Dental Team: An Additional Resource for Delivering Vaccinations

**DOI:** 10.3389/fmed.2021.653861

**Published:** 2021-03-25

**Authors:** Stefan Serban, Zhain Mustufvi, Jing Kang, Sally Eapen Simon, Siobhan Grant, Gail Douglas

**Affiliations:** ^1^School of Dentistry, University of Leeds, Leeds, United Kingdom; ^2^Public Health England, North East and Yorkshire Regional Office, Leeds, United Kingdom

**Keywords:** COVID-19, coronavirus, dentistry, dental team, vaccinations, influenza, flu, immunisation

In the original article, there was an error. The examples of countries where dentists can deliver flu vaccinations included New Zealand. This is a mistake as New Zealand should not have been on that list.

A correction has been made to the **Delivery Methods**, **Flu vaccinations**, **Subsection One-Stop-Shop**:

“Patients could choose to receive their influenza vaccine at the same time as their dental appointment. This model would support the principle of reduction of the number of contacts between patients and healthcare settings and reduce the risks associated with multiple journeys, especially in more rural areas where traveling longer distances is often required. It could be done both opportunistically as well as in a targeted manner for specific cohorts. This approach has already been successfully implemented in certain states in the US (13).”

Reference 23 was also removed from the reference list.

The authors apologize for this error and state that this does not change the scientific conclusions of the article in any way. The original article has been updated.
